# Digital autopsy teaching in pathology: Addressing overlooked gaps in implementation and evaluation

**DOI:** 10.1016/j.acpath.2026.100285

**Published:** 2026-08-07

**Authors:** Preeti Lamba

**Affiliations:** Dr. D. Y. Patil Vidyapeeth (Deemed to be University), Pimpri, Pune, Maharashtra, India

**Keywords:** Autopsy, Medical education, Digital learning, Pathology, Simulation

I read with great interest the recent article by Wraith et al. on the development of a narrated video autopsy for medical students.^1^ The authors present an innovative approach to address declining exposure to autopsy in undergraduate training. While this work provides valuable insights into modernizing pathology education, several important aspects merit further consideration.

First, although the study reports improved self-perceived understanding following the intervention, it does not incorporate an objective assessment of competency or long-term knowledge retention. While the authors acknowledge reliance on immediate self-reported outcomes, the absence of objective evaluation is not explicitly emphasized as a key methodological limitation. Evidence suggests that perceived learning may not reliably reflect actual competence, highlighting the importance of objective assessment strategies in educational interventions.^2^

Second, the study does not adequately explore the potential trade-off between digital learning and experiential exposure. As noted in the original article, autopsy provides important experiential learning in clinical reasoning and professional development, yet the implications of replacing this with virtual modalities are not discussed. Contemporary evidence indicates that autopsy-based learning remains essential but underutilized in medical education.^3^

Third, participation in the evaluation was voluntary, with variable response rates across survey items. However, the potential for selection or response bias is not addressed. Students with greater engagement or favorable perceptions toward digital learning may have disproportionately influenced the findings, thereby affecting the interpretation of effectiveness.

Finally, while the intervention demonstrates feasibility within a single institution, the study does not sufficiently address implementation challenges across diverse educational settings, including variability in institutional resources, infrastructure and curricular integration. Additionally, ethical considerations related to the recording, storage, and reuse of cadaveric material are only briefly described. Recent literature emphasizes the need for structured integration of autopsy teaching within medical curricula to ensure its continued relevance.^4^

In conclusion, Wraith et al.^1^ contribute meaningfully to advancing digital pathology education. Future work should incorporate objective outcome measures, address experiential limitations, evaluate potential biases, and provide deeper insight into ethical and implementation challenges to ensure effective and sustainable integration of such innovations.
